# Recombination between coronaviruses and synthetic RNAs and biorisk implications motivated by a SARS-CoV-2 FCS origin controversy

**DOI:** 10.3389/fbioe.2023.1209054

**Published:** 2023-08-04

**Authors:** Siguna Mueller

**Affiliations:** Independent Transdisciplinary Researcher, Kaernten, Austria

**Keywords:** DURC, recombinability, interactions between the natural and man-made world, coronavirus, biorisk spectrum, deliberate attacks, crime harvest, Trojan horse

## Abstract

The urgent need for improved policy, regulation, and oversight of research with potential pandemic pathogens (PPPs) has been widely acknowledged. A 2022 article in Frontiers in Virology raises questions, reporting on a 100% sequence homology between the SARS-CoV-2 furin cleavage site (FCS) and the negative strand of a 2017 patented sequence. Even though Ambati and collaborators suspect a possible inadvertent or intentional cause leading to the FCS insert, the related underpinnings have not been studied from the perspective of potential biorisk policy gaps. A commentary on their article contests the low coincidence likelihood that was calculated by Ambati et al., arguing that the sequence match could have been a chance occurrence alone. Additionally, it has been suggested that the odds of the recombination event may be low. These considerations seem to have put many speculations related to any implied viral beginnings, notably from a research setting likely outside the Wuhan Institute of Virology, to rest. However, potential implications for future disasters in terms of biosafety and biosecurity have not been addressed. To demonstrate the *feasibility* of the Ambati et al. postulate, a theoretical framework is developed that substantially extends the research orientations implicated by these authors and the related patent. It is argued that specific experimental conditions, in combination, could significantly increase the implied recombination profile between coronaviruses and synthetic RNAs. Consequently, this article scrutinizes these largely unrecognized vulnerabilities to discuss implications across the spectrum of the biological risk landscape, with special attention to a potential “crime harvest.” Focusing on insufficiently understood features of interaction between the natural and man-made world, vulnerabilities related to contaminants, camouflaging, and various misuse potentials fostered by the digitization and computerization of synthetic biology, it highlights novel biorisk gaps not covered by existing PPP policy. Even though this work does not aim to provide proof of the viral origin, it will make the point that, in theory, a convergence of under-appreciated lab experiments and technologies could have led to the SARS-CoV-2 FCS insert, which analogously could be exploited by various threat actors for the clandestine genesis of similar or even worse pathogens.

## 1 Introduction

The realization that an analysis such as the one below was necessary arose over a 2-year discussion with The European Union Agency for Cybersecurity which had been tasked with evaluating cybersecurity gaps in the life sciences (Mueller and Barros Lourenco, 2023) as fostered by the digitization of biology, computerized applications, and web-interfaces. This led to the question of applications, particularly in synthetic biology, which, when compromised, could have systemic implications and endanger critical infrastructure. These technologies may fall under the new European NIS2 Directive (The European Commission, 2023) which makes organizations engaged in such type of work compliant with the requirements necessary for the protection of critical infrastructures (Mueller and Barros Lourenco, 2023).

In this light, this article investigates specific regulatory and assessment gaps that arise from a controversy surrounding a postulated origin of the furin cleavage site (FCS) insertion into SARS-CoV-2. This particular topic is not analyzed to prove the beginnings of the virus, but rather, to highlight the feasibility of these controversial issues and the ensuing potentials for future malicious exploitation.

*Background:* The Covid pandemic has been one of the most destructive events in modern human history. Over 3 years since the first emergence of the new virus, there is still no consensus about its origin. While initially, a natural spillover event was essentially taken for granted (Andersen et al., 2020), studies and investigations by the Lancet Commission, the FBI, and an assessment by the Energy Department concluded with varying degrees of confidence that its likely source was an accidental release from the Wuhan Institute of Virology (WIV)^1^. In an April 18 bombshell release^2^, a report by the U.S. Senate Committee on Health, Education, Labor and Pensions asserts that SARS-CoV-2 likely resulted from an accidental leak at a laboratory in Wuhan.

In recent years, gain-of-function (GoF) work has been heavily criticized, and many leading experts, including former Centers for Disease Control and Prevention (CDC) Director Dr. Redfield, argue that even while such a type of work aims to prevent or prepare for a pandemic by artificially improving “the ability of a pathogen to cause disease”^3^, that, on the contrary, it “caused the greatest pandemic we’ve ever seen^4^.”

With certain coronaviruses (CoVs), it has long been known that the presence of a furin cleavage site (FCS) plays a key role in cell tropism and pathogenesis of these viruses. Over the years, this observation has triggered many research projects to better assess how the insertion of such cleavage sites into specific viruses could possibly enhance their transmissibility, expand their tropism, and increase their pathogenicity (reviewed in (Chan and Zhan, 2022)). A common observation has been that the insertion of an FCS has always made these viruses more dangerous. Significantly, while several CoVs do have an FCS, SARS-CoV-2 is the only member of the subgenus *sarbecovirus* with this characteristic. The finding of this unique FCS, absent among all SARS-related CoVs but inserted into SARS-CoV-2, has already rather early during the pandemic been seen as a significant piece of evidence to suggest that SARS-CoV-2 may be the product of laboratory manipulation (Chan and Zhan, 2022; Harrison and Sachs, 2022). Albeit, by just looking at the sequence, there is no way to determine whether humans or nature inserted this novel site into the virus (Cyranoski, 2020), which highlights but one difficulty of how “risky” research could be attributed, identified, and regulated.

The question as to what type of research should be regarded as “too dangerous” has triggered hefty discussions. For example, a recent policy analysis by a U.S. biosecurity panel found numerous loopholes and weaknesses in current regulation and oversight. Even though the panel agreed on a long-awaited set of recommendations, they are concerned about the vagueness of some of these, and in practice, the U.S. rules for risky pathogen research remain unclear (Reardon, 2023).

Specific research, such as experiments with an FCS, has previously been recognized as “too risky,” as evidenced by past expert advice. Notably, the insertion of FCS sequences into SARS-like viruses, a stated goal of the “DEFUSE” project, was in 2018 rejected by DARPA because the risks were deemed too high (Harrison and Sachs, 2022). While this does not exclude the possibility that such work was carried out using different funding sources, the research community is largely aware of potential perils associated with FCS research.

The key point raised here is that in addition to the debates surrounding the tradeoffs between the risks/benefits of inserting an FCS, or stated manipulations on the spike protein overall (Jocelyn, 2023), there may be yet another possibility for the emergence of the FCS altogether that has fallen outside regulatory oversight.

*Specific research that may escape regulation:* At the core of the ongoing controversy of what type of research should be restricted is that lab work is inherently dual-use (DU): the same research can be used for both benevolent and harmful purposes. Some DU work could potentially lead to disasters with far-reaching potential, known as DU research “of concern (DURC),” which, in a synthetic biology context essentially means that pathogens are being made even more dangerous^5^.

It will be suggested below that it may be possible that even research that does not have stated DURC/GoF risks (such as recognized for the FCS) may cause significant damage and even lead to a pandemic. Seemingly taboo topics in the context of biorisk assessment and management have been 1) that of well-intended research to engender adverse outcomes, and 2) that in a criminal context, benign R&D could be hijacked and have catastrophic consequences. Clearly, allegations that research or applications in synthetic biology that are widely regarded as beneficial could result in disasters nonetheless, would have significant disruptive effects across academia and industry, even if unsubstantiated. This makes it even more important that careful attention be placed on research efforts that fall outside of regulation, to help minimize the accidental or deliberate exploitation of any previously unrecognized gaps.

Concretely, this work scrutinizes specific aspects of CoV recombination in a laboratory setting. This is not done from the perspective of targeted viral mutations *per se*, for example, via direct mutagenesis or stated DURC/GoF work, but in the context of benign experiments such as in cancer research. More specifically, the starting point will be the discovery of (the reverse complement of) a proprietary sequence encompassing the SARS-CoV-2 FCS (Ambati et al., 2022), which will serve as a key example to investigate largely unrecognized gaps (for a summary of (Ambati et al., 2022), see Table 1 and Sect. 2).

**TABLE 1:** Poorly recognized biorisks which can be inferred from the scenarios suggested by Ambati et al. (2022): The finding of a purportedly proprietary sequence in SARS-CoV-2 encompassing the FCS, in the context of cancer research, raises many questions related to unrecognized biosafety and biosecurity dangers.

Key points made by Ambati et al. (2022)	Alternative views, main questions, and comments
Ambati et al. report on the presence in SARS-CoV-2 of a 19-nucleotide RNA sequence	Although the beginnings of COVID have not been unambiguously established, the main hypotheses include either a natural genesis (zoonosis (Andersen et al., 2020; Calisher et al., 2020)) of the virus, or a laboratory origin (in the context of viral and GoF research). The hypothesis by Ambati et al. (2022) raises the potentiality for a radically different laboratory origin, even if of the SARS-CoV-2 FCS alone, that is outside the scope of viral GoF/DURC policy and regulation
• The novel insert encompasses and encodes the novel FCS of its spike protein	
• It has 100% identity to the reverse complement of a proprietary MSH3 mRNA sequence (identified as SEQ ID11652, nt 2751-2733, see below)	
• The insert could have happened during laboratory research via some recombination event	
• Copy-choice recombination could have been realized during cancer research via infection of SEQ ID11652-MSH3-transduced human cells by a SARS-like virus	The explicit goal of the Moderna patent (Bancel et al., 2017) is to enhance cancer research. However,
• This proprietary sequence (SEQ ID11652) is found in a US patent filed by Moderna on Feb. 4, 2016 (Bancel et al., 2017)	• *A priori*, the motivation for combining human cancer research with SARS-based viral research is not clear
• Specifically, the sequence listing in US9587003B2 revealed an artificial sequence fragment comprising 5′-CTA​CGT​GCC​CGC​CGA​GGA​G-3’ (nt 2733-2751 of SEQ ID11652). The corresponding mRNA would have 3′- GAU​GCA​CGG​GCG​GCU​CCU​C - 5′, or equivalently, 5′- CU CCU CGG CGG GCA CGU AG - 3,’ which is a 100% match to the original SARS-CoV-2 strain from Wuhan (ntds 23547-23565 in the SARS-CoV-2 genome), in which the four codons CCU CGG CGG GCA exactly yield the PRRA furin cleavage site	• Even though Ambati and collaborators suspect an inadvertent or intentional act during the course of viral research, the odds of the implied recombination event could be low
According to Ambati et al. (2022), the reason for using MSH3 may have been that	The context of such viral experiments is not clear at the outset. MSH3 is a human DNA repair gene
• Overexpression of MSH3 is known to interfere with mismatch repair	
• Mismatch repair deficiency could have been important during the research with SARS-like viruses	
Accordingly, Ambati et al. propose the following mechanism leading to the integration of the novel sequence surrounding the SARS-CoV-2 FCS	This specific research context raises several immediate questions
• Human cell lines may have been transfected with MSH3	• Is there a rationale for conducting research that combines a) DNA repair pathways, b) induction of DNA repair deficiency, c) CoV research involving SARS-like viruses, and d) cancer research (the goal of the patent)?
• This could inadvertently or intentionally have induced mismatch repair deficiency	• What is the potentiality of CoV evolution/escape via recombination in such a research context (deliberate or accidental)?
• Such cells co-transfected with a SARS-like virus (expressing appropriate enzymes such as RdRp) could have led to copy-choice recombination between the MSH3 and the virus	• Could a specific experimental framework increase the odds of the postulated recombination event?
The actual proprietary sequence does not represent the corresponding sequence surrounding the FCS in SARS-CoV-2, but its reverse complement. Hoverer, according to Ambati et al. (2022)	From a biorisk perspective, this is critical
• Single stranded RNA viruses such as SARS-CoV-2 utilize negative strand RNA templates in infected cells	• Dangerous sequences could effectively be camouflaged by their harmless-looking reverse complement
• Copy choice recombination with a negative sense SARS-like RNA could have led to the integration of the MSH3 negative strand	• Via this concealment, the dangerous sequence itself would not be detected during screening
• That is, recombination of the sequence may have happened despite being on the opposite strand of the open reading frame	• Such camouflaging is a substantial vulnerability to both unintended mishaps and deliberate forms of misuse
	• Specifically, the integration of short fragments from antisense strands has been observed in experimental models (see (Ambati et al. (2022) for references)

Explicitly, Ambati et al. (2022) identify an unexpected relationship between this new sequence insert in SARS-CoV-2 and a previously patented sequence. More precisely, they obtain the coincidence probability for the occurrence of the sequence homology between the new FCS insert in the SARS-CoV-2 genome and (the negative strand of) the patented sequence as 3.21 × 10^−11^. This extremely low number is the basis of their conclusion that this represents a “highly unusual” phenomenon, asking for potential explanations for this correlation which should be further investigated.

**FIGURE 1:**
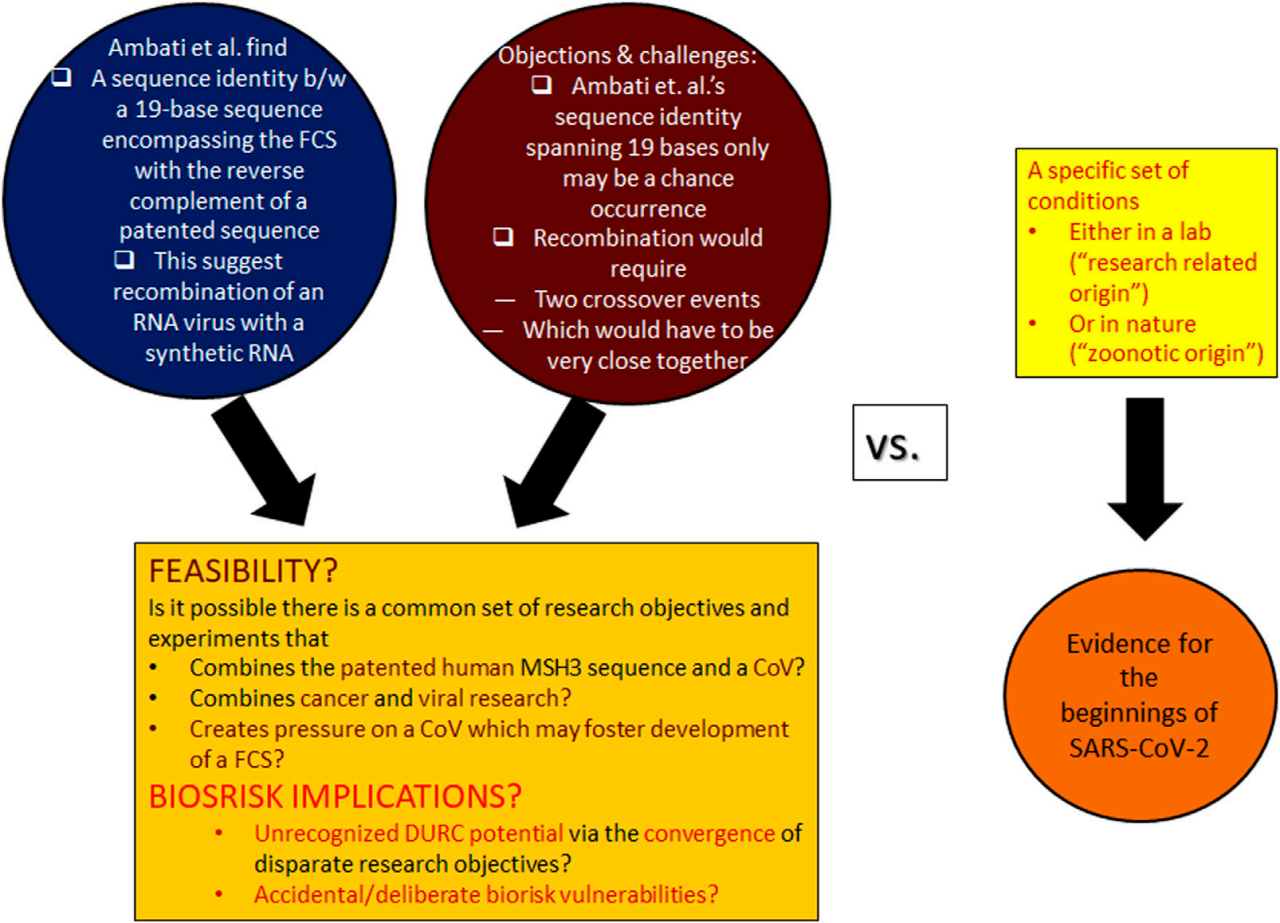
Postulated presence of the negative strand of a patented sequence in SARS-CoV-2, the feasibility of chance homologies, and implications to inform biorisk assessment. Left: The main focus of the article by Ambati et al. (2022) was to draw attention to the critical sequence insert surrounding the SARS-CoV-2 FCS, which, according to their analysis, is 100% complementary to the negative strand of a previously patented sequence. In Ref. Ambati et al. (2022), the authors also calculated, and obtained, a very small probability for this unexpected sequence homology. They also gave a very basic framework of how the purportedly patented sequence could have been integrated into a SARS-like virus, but they leave several questions about the rationale of such a research entertainment unanswered. Additionally, it has been suggested that the specific recombination event may happen very rarely, and, furthermore, the calculated coincidence probability has been contested and published as a Commentary (Dubuy and Lachuer, 2022) to Ref. (Ambati et al., 2022). Against this backdrop, the analysis undertaken here investigates the feasibility of the implied viral recombination event and consequential biorisk dangers with special attention to deliberate misuse potentials for future exploitation. It is important to contrast the current analysis, which asks if/how specific laboratory experiments could have led to the special insertion in SARS-CoV-2 or other recombination events, from the actual investigations of the beginnings of SARS-CoV-2 (right).

Their extremely low coincidence probability has been contested. A commentary (Dubuy and Lachuer, 2022) to Ref. (Ambati et al., 2022) questions the BLAST search conducted by Ambati et al. as well as how the probabilities were calculated. Furthermore, the recombination between a CoV and a synthetic RNA as suggested in Ref. (Ambati et al., 2022), requiring two crossover events that would have to be very close together, may be regarded as practically unlikely^6^. These constraints and the counterargument offered in (Dubuy and Lachuer, 2022) that the observed sequence homologies may just be a chance occurrence seem to have put the question raised in (Ambati et al., 2022) to rest. However, these developments and controversies have not been sufficiently scrutinized. The genetic recombination envisioned by Ambati et al., if proven feasible, has grave biorisk implications for future events (Figure 1).

It is important to note that biological risk covers a spectrum encompassing naturally occurring, unintended, and deliberate risks (The Royal Society and the International Council for the Life Sciences, 2009). Furthermore, adding to the difficulty of distinguishing natural from man-made risks, as seen in the ongoing Covid origin debate, the analogous problem applies to parsing out unintended from deliberate events. In the context of increased reliance of synthetic biology on technology, separating safety (which focuses on vulnerabilities fostered by unintentional issues) from security (which targets deliberately induced vulnerabilities) may not be easy (Mueller, 2020). Now, as there is no sound rationale that supports the notion that SARS-CoV-2 was intentionally released from a lab, past origin discussions have not paid special attention to factors that could be deliberately misused. However, looking ahead, it is important to additionally scrutinize security aspects as well. As with all emerging technologies, a failure to do so may lead to a scale of exploits that previously has been called a “crime harvest” (Pease, 1997; Elgabry et al., 2022).

*Key open questions to be addressed:* This article first investigates the very feasibility of the emergence of the FCS as suggested by Ambati et al. or via related experiments. It then scrutinizes the resulting vulnerabilities, across the full range of the biorisk landscape, ranging from unintended accidents to deliberate malicious exploitation.

Just as the actual beginnings of SARS-CoV-2, due to a lack of early data, are to a large part limited to a rational investigation, the analysis conducted here builds on logical deduction. The main questions analyzed below are the following.

• Even though Ambati et al. suggest that the novel insert in SARS-CoV-2 could have come about inadvertently or intentionally, the odds of the implied recombination event may be low. Notwithstanding this, is there a logical rationale for some rather feasible experimental underpinnings that could substantially increase these odds?
• Is it possible that the disparate research goals implicated in Ref. (Ambati et al., 2022) (research with SARS-like viruses) and the patent (Bancel et al., 2017) (cancer research) can be reasonably extended so that whilst neither includes the integration of an FCS into a CoV, they could have converged into one joint set of laboratory experiments enabling its integration nonetheless?
• If the likelihood for the implied sequence homologies is as high as argued in (Dubuy and Lachuer, 2022), what does this mean in terms of biorisk potentials, especially regarding deliberate exploitation?
• What do these factors *combined* mean from an existing biorisk management perspective, especially related to crime risks and deliberate attacks?

*Outline:* The article begins with a description of the postulated genesis of the FCS in SARS-CoV-2 from a laboratory context as suggested in (Ambati et al., 2022). It continues with a review of the feasibility and orientation of the implicated research objectives, as well as those extended below, and argues that these could have aggregated in a unifying research project which may have favored viral evolution and escape and, consequently, recombination with synthetic RNAs as envisioned by Ambati et al. It then highlights challenges with existing biorisk policy, placing special focus on deliberate attack potentials. Finally, it concludes with a summary and recommendations.

## 2 Viral research in the context of cancer research: an analysis of the feasibility of the Ambati et al. postulate

This section gives a brief introduction to the postulated route of how the SARS-CoV-2 FCS could have evolved as first suggested in (Ambati et al., 2022). Given that the implied research setting, along with their apparently vastly disconnected features, may not have favored the particular viral recombination in question, additional rational research aims and contexts are identified that could provide the necessary framework and substantially increase the chance of such events.

### 2.1 Synopsis of the hypothesis by Ambati et al. concerning the proprietary sequence in SARS-CoV-2 and main open questions

As mentioned, in early 2022, a publication in Frontiers in Virology (Ambati et al., 2022) described an intriguing finding. First, Ambati and collaborators note that among numerous point mutation differences between SARS-CoV-2 and the bat RaTG13 CoV, only the 12-nucleotide FCS exceeds 3 nucleotides. During the pandemic years, the FCS has been regarded as one of the most, if not *the* most important novel characteristics of SARS-CoV-2. CoVs, just as RNA viruses in general, are subject to numerous random point mutations. Given the high error rate of the RNA replicase and the very structure of the virus genome itself, it is unclear how a random insertion mutation could explain the emergence of the FCS without substantial additional changes throughout the genome (Romeu and Ollé, 2021).

Intriguingly, specifically related to the SARS-CoV-2 FCS, Ambati et al. (2022) report on a BLAST search for the 12-nucleotide insertion which, surprisingly, revealed a 100% reverse match in a Moderna patented sequence listing (SEQ ID11652) in US patent 9,587,003 filed on Feb. 4, 2016 (Bancel et al., 2017). Furthermore, according to Ambati et al., an examination of SEQ ID11652 showed that the match extended beyond the 12-nucleotide insertion to a 19-nucleotide sequence that encompasses the FCS (Table 1).

The reverse complement sequence present in SARS-CoV-2 may occur randomly. However, Ambati and collaborators report that the artificial 19-nucleotide sequence fragment is without precedence in any mammalian or viral genome in the BLAST database except in SARS-CoV-2.

Ambati et al. also point out that the unprecedented sequence encompassing the FCS not only is a 100% complementary match to the Moderna proprietary sequence in (Bancel et al., 2017); furthermore, SEQ ID11652 is transcribed to the human mutS homolog (MSH3), which they think is codon optimized for humans.

The aim of the Moderna patent in question, titled “Modified Polynucleotides For The Production Of Oncology Related Proteins And Peptides,” is cancer treatment. Specifically, it “relates to compositions and methods for the preparation, manufacture and therapeutic use of oncology-related polynucleotides, oncology-related primary transcripts and oncology-related mmRNA [modified mRNA] molecules.” In line with this, Ambati and collaborators suggest that MSH3 replacement with a codon-optimized mRNA sequence for human expression likely has applications in cancers with mismatch repair deficiencies. More specifically, this leads to their far-reaching hypothesis: they postulate that a specific recombination event during cancer research may have led to the integration of the MSH3 negative strand, including the FCS, into the genome of a precursor of SARS-CoV-2, leading to the novel FCS.

Concerning the postulated occurrence of the patented sequence in the SARS-CoV-2 genome, the identified sequence is on the opposite strand of the open reading frame in SEQ ID11652. Nonetheless, Ambati et al. provide a mechanistic explanation that captures the molecular underpinnings to resolve this apparent limitation. Single-stranded RNA viruses such as SARS-CoV-2 utilize negative-strand RNA templates in infected cells. Ambati et al. suggest that the artificial 19-nucleotide sequence present in the human MSH3 gene might have been introduced into the SARS-CoV-2 genome through copy choice recombination with a negative sense SARS-CoV-2 progenitor RNA in infected human cells. If so, this would imply that from a biorisk perspective, homologies and recombinations between pertinent strands as well as those involving their complements need to be taken into consideration.

The above raises numerous questions which will be further analyzed below.

• Feasibility: Is the scenario hypothesized by Ambati et al. theoretically feasible?
• The reasons why Moderna could have engaged in particular experiments: specifically, is there a legitimate reason why/how to combine cancer research, viral research, and synthetically modified mRNAs?
• Genetic recombination: The postulated recombination would have required two crossover events. As the novel insert comprises only 19 nucleotides, these crossovers would have to be very close together, which some may think makes the frequency of such events very low. Nonetheless, is it possible that specific laboratory settings that exploit the natural recombinability of CoVs and other unrecognized properties, increase the odds of such a genetic recombination event?
• Misuse: What is the practical feasibility to maliciously exploit the implicated and related gaps?
• What do sequence homologies, especially if they are rather likely as argued in (Dubuy and Lachuer, 2022), mean for laboratory safety and security, and in particular, those where both the original as well as the reverse complement may be of biological relevance (e.g., with MSH3 and FCS, see below)?

### 2.2 CoV recombination - insights from decades-old research

CoV recombination has been increasingly investigated since the Covid pandemic. However, the focus of past studies has mainly been that of inferring natural evolution and relationships between SARS-CoV-2 and its potential progenitors. For instance, as done in (Yang et al., 2021) via a detailed automated analysis, such type of investigation relies on the number and diversity of representative CoV genomes available, and therefore cannot directly predict recombination characteristics between CoVs and synthetic RNAs, and even less so, fostered by enhanced laboratory evolutionary pressure. In the following, therefore, the analysis places special focus on well-established recombination characteristics that lend themselves to the situation postulated by Ambati et al.

RNA recombination was first identified in the early 1960s as an exchange of genetic material between closely related RNA viruses. CoVs, in particular, have long been known to utilize RNA recombination, possibly because of their large genome size and the large number of errors during RNA replication. Indeed, already in 1996, Lai discovered that for mouse hepatitis virus (MHV) not only was recombination frequency high. Many of the recombinants had even multiple cross-overs. Furthermore, the recombinant viruses grew at non-permissive temperatures and became the predominant virus population after only two tissue culture passages. The only explanation for this was that recombinant viruses had evolutionary advantages over parental viruses under experimental conditions (Lai, 1996).

For at least 20 years now, precise details for a variety of RNA recombination events have been clearly established (Chetverin, 1999). In the context of the possible genesis of the SARS-CoV-2 FCS, as postulated in (Ambati et al., 2022), several features of RNA recombination are worth mentioning.

• Already in 1996, it was suggested that CoVs in particular may utilize RNA recombination to counter the possibly deleterious effects of their high mutation rate. In fact, at that time it was already well-established that CoVs undergo recombination at a very high frequency of nearly 25% of the entire genome.
• Already in the 1970s, it was known that special “defective interfering (DI) particles” (previously known as “inactive viruses” - so called because they lacked some viral genes) were able to propagate following some non-homologous RNA recombination events. Importantly, the components could be identified that made it possible to provide the missing proteins in *trans*: they then could be detected as particles contaminating the virus preparation (Chetverin, 1999).
• Ref. (Rowe et al., 1997) established 25 years ago that for CoVs, recombination can also happen during passage in tissue culture. This establishes the very basis that the proposed recombination event postulated by Ambati et al. (2022) was not the result of slow adaptations among naturally occurring viruses but indeed could have been realized in a laboratory context.
• Additionally, in 1997, Rowe et al. (1997), specifically analyzing the spike protein of MHV to study spike deletion variants, found that RNA recombination can occur during either positive or negative strand synthesis - thereby supporting the suggestion offered by Ambati and collaborators of the recombination event involving negative sense RNA intermediates.

In general, the generation of a recombinant sequence can mechanistically be conceived in two different ways (Chetverin, 1999): 1), via breaking the parental sequences and joining the resulting fragments, or 2) via *de novo* synthesis by the viral replicase which switches to another template after it has copied a portion of the first template. Notably, already more than two decades ago, details and refinements of these were known.

• Back in 1999, one of the most surprising discoveries was that of RNA *self*-recombination. This means that RNA molecules can recombine without any DNA intermediates. Precisely, self-combination is a general property of RNA, requiring nothing but RNA itself and Mg2+. Chetverin (1999) concluded that it must be ubiquitous in nature and involve both viral and cellular RNAs. (By extension then, the same could also apply to lab experiments and also synthetic RNAs.)
• Apart from RNA recombination performed by, and as an inherent feature of, RNA itself, recombination is also known to be promoted by some proteins. These include replicase- (more below) and ribozyme-assisted recombination (Chetverin, 1999).
• The template-switch mechanism for RNA recombination has been demonstrated in two other interesting contexts. Both were initially believed to not be available for (natural) RNA viruses but may be especially relevant in the context of lab experiments.
  (i) The first involves retroviral reverse transcriptases. Notably, Negroni et al. (1995) showed that Moloney murine leukemia virus reverse transcriptase (RT) alone promotes homologous recombination efficiently. The important point they are making, for lab experimentation in particular, is that while RNA concentration itself has little effect on recombination frequency, there is a clear correlation between the amount of RT used in the assay and the extent of recombination observed.
  (ii) Furthermore, for RNA recombination, the ‘switching between template’ mechanism has also been directly demonstrated for DNA polymerases during PCR (Chetverin, 1999; Innis et al., 2012).
  (iii) Chetverin (1999) believes that both the mechanism via the retroviral RT activity and PCR assist the template-switch mechanism in that they enable dissociation of the nascent strand base-paired to its template. In the former case, the RT has an inherent RNA template degradation mechanism, and in the latter, this is realized during the heat-induced melting of the DNA duplexes. As pointed out by Chetverin, RNA viruses may have evolved analogous mechanisms to overcome the duplex problem. It seems feasible that the repeated melting during either PCR or RT-PCR under laboratory conditions may help dissociate the nascent RNA strand from their template. In addition, while not a focus of ref. (Chetverin, 1999), with CoVs, the mechanism of the viral polymerase itself is now known to facilitate that step very efficiently (more below).

### 2.3 Recombination in CoVs and basic links between cancer and viral research that could support the Ambati et al. hypothesis

The fact that CoVs are very amendable to recombination has been known for decades. Pivotal work in this regard was first obtained by Lai (1996) who argued that for CoVs, due to their large genome size, recombination is a valuable tool for virus evolution, to counter the large number of errors made during replication, but also to provide diversity in genomic structure and hence, offer evolutionary advantages for recombinants under specific conditions (including experimental).

The important point is that CoV evolution may thereby not happen by a slow accumulation of adaptive mutations in a piecemeal fashion, as has been the basis of substantial pandemic research on the origin of SARS-CoV-2—which has often centered on individual ntd changes and sequence-based measures and determinants but which may not be the most optimal (Piplani et al., 2021). A notable exception is a paper by Gallaher (2020) who proposed that RaTG13, a relatively recent ancestor of SARS-CoV-2, likely experienced a number of sudden changes which can be explained, he argues, by several recombination events.

The usefulness and potential of recombination in the lab were already known in 1999 when Lai described how RNA recombination could be utilized to achieve desirable consequences for CoV studies. Notably, the setup is exactly the same as postulated in (Ambati et al., 2022): the basic step consists of manipulating certain mRNA constructs which are then transfected into virus-infected cells. Based on several success stories in the lab, Lai concludes that this approach is very useful for introducing certain sequences into viral RNA. He concludes that, given the limitations of CoV research at that time (owing to their size), such a “recombination strategy provides an alternative method for introducing site-specific mutations into the viral genome.”

Research has significantly advanced during the last few decades. It is not clear to what extent Moderna was, or was not, attempting to use viral recombination for research purposes. From a theoretical perspective, a key question is why/how cancer research could have been linked to viral evolution. It seems feasible that in a laboratory context, Moderna (Bancel et al., 2017) attempted to do a combination of approaches. These are discussed in greater detail below for their underappreciated potentials related to biorisk assessment and mitigation and not to imply any culpability of Moderna related to the origin of SARS-CoV-2.

• *mmRNAs as therapeutics against viruses implicated with cancer*: Central to Moderna’s patent is the use of novel (modified) mRNAs that Moderna aimed to deploy as therapeutic agents. Since viruses can compromise human health at many levels, and have been implied with the development of various cancers as well (Zapatka et al., 2020), it seems feasible that Moderna may have tested various synthetic mRNAs as therapeutic modalities in the context of various viral infections and viral variants.
• *Accidental (unintended) viral modification*: The development of therapeutics against certain cancer-implicated viruses would likely have involved viral mutagenesis. During the last few decades of viral research, prominent methods have emerged such as synthetic genomics techniques which have even enabled the rapid reconstruction of SARS-CoV-2 from synthetic DNA (Thao et al., 2020), and the focus may have shifted away from targeted RNA recombination. Thus, Moderna may not have been sufficiently aware that their research setup (Bancel et al., 2017) mimicked the very experimental conditions of Ref. (Lai, 1996) to influence viral evolution in the lab. Given that the mechanism of recombination may create new viruses, this may have happened unexpectedly, e.g., via contaminated or accidently switched cell lines infected with some SARS-CoV-2 precursors.
• *Targeted viral mutagenesis to study cancer-causing viruses and their susceptibility to the therapeutic agents*: As mentioned, in 1999, Lai described some success stories of CoV research of how recombination was able to replace some previously “defective genes” in specific CoVs. That is, when specific RNA fragments were transfected into cells infected with a mutant carrying a defective N gene, recombinant viruses with a functional N gene were obtained. In similar events, RT-PCR confirmed the presence of the transfected RNA fragment. It seems feasible that Moderna may have used recombination as one of the means to gain new insights regarding CoVs and their cancer-causing properties. Additionally, the aim may have been to assess viral survival and evolution when in the presence of the new therapeutic mmRNAs.
• *Viral mutagenesis for the development of recombinant CoVs as a cancer vaccine and tested in cells transfected with mmRNAs*: Rather than developing certain mmRNAs as therapies against cancer-causing viruses, CoVs themselves may have been analyzed for their potential as a vector to deliver the therapeutics, and tested in susceptible human cells (e.g., those over-expressing MSH3 to induce DNA repair deficiency). CoVs may have been of great interest as they represent an RNA virus that was long believed to be unable to integrate into the host genome (but see below).

### 2.4 Feasibility of integration of a short stretch of synthetic RNAs into a CoV

As stated, it has been known for decades (Lai, 1996; Rowe et al., 1997; Graham and Baric, 2010; Gallaher, 2020) that the mechanism of recombination in RNA viruses is template switching. In this case, recombination takes place during RNA replication, i.e., when the RNA polymerase pauses at certain sites of the RNA template. As first postulated by Lai (Lai, 1996), the nascent RNA transcripts separate from the original template, and then join themselves to a different RNA template to continue RNA synthesis.

From this perspective, one of the key questions that remain to be addressed when assessing Ambati et al.’s hypothesis is: how is it possible that only a short stretch of 19 ntds was integrated into a SARS-like genome even though during the experiments, cells would have been transfected with the full-length sequence that codes for MSH3? That is, why is it that transfection did not result in the integration of the full sequence, and instead, just included the short 19-ntd part including and surrounding the FCS alone? Interestingly, intrinsic features of CoV transcription itself may explain, theoretically, at least, how this could have happened.

• Notably, for CoVs, mRNA transcription is done in a discontinuous manner (Lai, 1996; Rowe et al., 1997), with the viral polymerase functioning in a piecemeal fashion rather than progressing the entire viral genome at once. This fact is well established, as summarized by a recent publication by the NIAID (Sattar et al., 2023): “These coronaviruses contain a positive-strand RNA genome with a few unique features: two-thirds of the viral RNA is translated into a large polyprotein, and the remainder of the viral genome is transcribed by a discontinuous transcription process into a nested set of subgenomic mRNAs.”
• Necessary for the above discontinuous mechanism is that the viral polymerase and nascent RNA transcripts disassociate from the RNA template regularly during RNA transcription, and by necessity then, the CoV polymerase must jump between different RNA molecules during RNA synthesis (Lai, 1996).
• The realization that the CoV polymerase is not acting in a progressive manner is essential also for recombination - which is reminiscent of the disassociation from, and rejoining to RNA templates during mRNA transcription (Lai, 1996). Likewise, then, the RNA polymerase complex may jump to a spatially proximal template, and thus by falling off and rejoining, contribute to RNA recombination.
• Importantly, however, recombination is not a totally random event. Recombinants with chimeric viral proteins derived from different parental viruses are often unstable and have inferior replication ability. Noting that some cross-over sites were hardly detected among mutants of mouse hepatitis virus strains, Lai (1996) suggests that for recombination, certain cross-over sites appear to be restricted. He postulates that some aberrant recombination events would render the recombinants not viable under selection pressure and that for optimal viral growth, recombinants are favored that reflect specific viral RNA or protein structure requirements.
• Rowe et al. (1997) also determined that the functioning of the RNA polymerase, including its fragmented way of operation, is significantly dictated by specific secondary structures. Concerning the copy-choice mechanism of recombination, it was therefore long believed that recombination will occur frequently at RNA sites of strong secondary structure, based on the observation that these structures promote transcriptional pausing (Mills et al., 1978).
• Recent years have shown that recombination is a promiscuous event that is not significantly influenced by any single factor. Notably, in 2020, Alnaji et al. (2022) argued that recombination in positive-sense RNA viruses is not influenced by RNA structure, or even the RNA donor or acceptor sequence. Instead, they posit that genome function and fitness are of greater importance in determining the identity of recombinant progeny. This seems to contradict previous studies that emphasize the role of RNA structure, sequence identity, and the amount of base pairing in the donor and acceptor sequence (Zúniga et al., 2010; Sola et al., 2011), and may reflect variation in the recombination processes involved between different viruses or the involvement of host factors (Wang et al., 2022).
• Consequently, then it seems that both CoV RNA sequence and structural factors as well as selective pressure are responsible for recombination, with the former contributing to bringing particular regions of the RNA molecule in sufficiently close proximity, and the latter enabling the selection of propagation of more advantageous recombinants.

These underlying features enabling recombination seem important for the genesis of the patented sequence in SARS-CoV-2 as proposed by Ambati et al. (2022). They provide the rationale for the RNA polymerase to jump to a spatially proximal template and to come off after a short stretch. As highlighted by more recent research, RNA viruses undergo frequent and continuous recombination events over a prolonged period of time and favor the selection of the fittest recombinant genome (Bentley and Evans, 2018; Wang et al., 2022). This could explain the selection and retention of specific variants, e.g., those with the short insert that constitutes and encompasses the novel FCS. The hypothesis of lab-imposed selective pressure as a key factor to support such as recombination event will be further analyzed below where this will be linked to particular research experiments that would have made sense in the context under consideration, and which may have resulted in a new nuclear localization signal (NLS) that happens to be an FCS.

## 3 A potential framework that increases the odds of the genetic recombination as envisioned by Ambati et al.

Even though Ambati and colleagues believe that accidental or deliberate acts may have led to the viral recombination resulting in the new genetic insert in SARS-CoV-2 (Ambati et al., 2022), this section envisions a more detailed framework that could also increase the odds of such recombination events.

### 3.1 Cancer research, host DNA repair, and potentials for viral recombination

From a logical perspective, the postulated sequence insert in SARS-CoV-2, essentially identical to a patented sequence, may seem difficult to grasp since the patent in question targets cancer research in humans. How could this possibly be linked to viral research so that MSH3-transfected cells, then infected with a SARS-like virus, could have resulted in genetic viral recombination? This section analyzes potential research objectives of how such apparently disconnected issues could converge, and under which settings the odds for such type of recombination could be substantial.

#### 3.1.1 Disruption of DNA repair by DNA and RNA viruses

Viruses are responsible for various human health challenges including serious forms of disease. Some viruses introduce DNA damage and genetic instability in host cells during their lifecycles. Notably, some have been found to manipulate components of the DNA damage response (DDR), a network of complex mechanisms for DNA damage detection and repair to combat DNA damaging agents. Surprisingly, these include RNA viruses as well, even for those species where viral replication takes place exclusively in the cytoplasm. As detailed in (Ryan et al., 2016), by impairing DDR pathways, the resulting DNA damage can be a crucial component of the pathogenicity of RNA viruses, e.g., through the triggering of apoptosis, stimulation of excessive inflammatory immune responses, and the introduction of deleterious mutations in infected cells. The latter, in turn, will likely increase the risk of tumor development.

Since cancer research was one of the main components of the Moderna patent, this relationship between RNA viruses and tumor development may be one common denominator to explain and further refine the apparently disparate research components implicated by Ambati et al. (i.e., CoV research and DNA repair deficiency).

Specifically, Ryan et al. (2016) describe various key mechanisms during the RNA virus lifecycle and how they can induce genetic instability. Even though the exact source of DNA damage and consequences of DDR (de)activation are still unresolved, it is now clear that specific viruses are believed to derive some of their most pathogenic features, including tumorigenesis, through such cellular transformation mechanisms.

In the context of viral-host interactions, numerous questions may have triggered the attention of Moderna^7^.

• Mechanisms of how RNA viruses can trigger, influence, or impair DDR pathways: Although a common feature of DNA viruses, it has been known for some years now that also for some RNA viruses, it is frequently the case that viral proteins are often transported to the nucleus (Leon et al., 2012; Ryan et al., 2016). Once in the nucleus, they can obviously perturb various critical cellular functions, including the antiviral response; albeit, details of these mechanisms remain poorly understood.
• Nuclear transport involving CoVs: In 2016, when examining the potential of various RNA viruses, or some of their proteins, to be transported to the nucleus, it became clear that these also include common cold viruses including CoVs. Specifically, Ref. (Ryan et al., 2016) details how the Infectious bronchitis virus (IBV), a highly infectious avian CoV, may impair specific DNA damage signaling pathways and induce DNA replication stress, including via its interaction with DNA polymerase *δ* and modulation of cell cycle progression. Thus, comprehending key features of CoVs that enable their nuclear transport would be essential from both a scientific and public health perspective.
• Molecular mechanisms involved in the DDR: The DNA Damage Response and DNA repair pathways comprise a highly coordinated network of proteins that are activated in the presence of DNA damage, compromising a host of sophisticated mechanisms to deal with single- and double stranded DNA breaks. One of the most famous involves the cell cycle checkpoint protein p53, the guardian of DNA which promotes cell cycle arrest to prevent the replication of damaged DNA. Repair of single-strand DNA damage is realized via several repair pathways, *inter alia* via various MSH complexes (MutS*α* or MutS*β*). This seems highly relevant, as the sequence implicated in the Moderna patent (MSH3) is part of the MutS*β* complex (an MSH2-MSH3 heterodimer) which is involved in tumorigenesis through the maintenance of chromosomal stability. Importantly, both loss of expression and over-expression of MSH3 can lead to tumorigenesis (Marra et al., 1998; van Oers et al., 2014). On the other hand, the involvement of MutS*β* has been extensively studied in the context of severe genetic neurological disorders.

The following provides further details on why and how the above is relevant to the Ambati et al. hypothesis.

#### 3.1.2 Nucleocytoplasmic trafficking of viral proteins - an underappreciated target for antiviral therapy

Classically, it has been recognized that molecules larger than 45–50 kDa generally require specific amino acid sequences known as nuclear localization signals (NLSs) to gain nuclear entry (Leon et al., 2012). More precisely, nuclear protein import requires the recognition of the NLS-signal containing cargo proteins by members of the importin (IMP) superfamily of nuclear import receptors on the cytoplasmic side of the nuclear pore complex (NPC). After the transport complex docks to the NPC, it is translocated to the nucleus through the central pore; consecutively, once it is within the nucleus, the transport complex dissociates to allow the cargo to perform its nuclear function. Nuclear protein export occurs in an analogous fashion, where nuclear export signals are recognized by exportin proteins (Kylie et al., 2011; Leon et al., 2012).

As mentioned, the fact that viruses can facilitate the nuclear import and/or export of viral proteins in infected cells likely benefits viruses to carry out many functions ranging from essential replication activities such as DNA replication (DNA viruses), RNA synthesis (even for some RNA viruses such as Influenza A where synthesis of viral ribonucleoprotein complexes takes place in the nucleus (Ryan et al., 2016)), to the dampening of the host cell immune responses (Leon et al., 2012).

Some 10 years ago, this observation triggered the idea to specifically target the transport of specific viral proteins into the host cell nucleus as a therapeutic strategy (Leon et al., 2012). The inhibition of nuclear trafficking of viral proteins was recognized as an attractive possibility not only for retroviruses but also for many other RNA viruses which, despite their replication occurring in the cytoplasm, nonetheless transport some of their key proteins to the nucleus and thereby impair essential host processes.

The potential of preventing nuclear protein import seemed to be validated by some early studies that showed promises as potential therapies against HIV-1 and dengue by the recognition of a broad-spectrum inhibitor of the nuclear transport receptor importin *α*/*β* (Kylie et al., 2011; Leon et al., 2012). Specifically, for the dengue virus (DENV) which replicates in the cytoplasm and with no requirement for its genome to enter the nucleus, the nonstructural protein 5 (NS5), which serves as the viral RNA polymerase, is predominantly found within the nucleus of infected cells. Strikingly, in 2011, Kylie et al. (2011) demonstrated that inhibiting NS5 nuclear import using ivermectin, “a general inhibitor of IMP*α*/*β*1-dependent nuclear import,” was found to greatly reduce virus production, supporting the potential of targeting nucleocytoplasmic trafficking for therapeutic interventions.

#### 3.1.3 The targeting of nuclear import/export of viral proteins - general objectives.

Conceivably, these consist of the following.

• *CoV research to better comprehend details related to nucleocytoplasmic trafficking of viral proteins, including their consequences in the host.* Studies that have investigated the import of viruses or their proteins have traditionally heavily relied on mutagenesis, to e.g., express specifically mutated viral proteins or even created new viruses altogether. For example, Ozawa et al. (2007) report on the creation of a new influenza-A virus whose nucleoprotein contains amino acid substitutions to abolish its nuclear localization function; doing so helped identify specific viral NLSs that are essential for viral transcription and translation. For HIV-1, it is well established that this virus makes use of multiple import pathways under diverse conditions and in different cell types (Leon et al., 2012). On the other hand, for CoVs less seems to be known in this regard. Trying to comprehend inhibitors of nuclear import or export would likely have involved the transfection of viral proteins or susceptible/mutated viruses into human cells to study the interaction of key human and viral proteins involved in this process.
• *Identification of new drugs able to inhibit the import of viral proteins.* As noted above, in 2011, Wagstaff et al. (Kylie et al., 2011) developed a screening assay for the identification of specific inhibitors of nuclear import. In their case, using the HIV-1 integrase (IN) and importin (IMP) *α*/*β*1 interaction as a proof-of-principle, they were able to validate the activity and specificity of mifepristone and ivermectin to inhibit nuclear protein import in HeLa (human cervical adenocarcinoma) cells. The IMP *α*/*β*1 pathway is utilized by many RNA viruses, including SARS-CoV-2. Specifically, *in-vitro* studies (Caly et al., 2020) have confirmed that ivermectin is able to bind to and destabilize the IMP *α*/*β*1 heterodimer and thereby prevents viral proteins from entering the nucleus. It would have made sense to try to extend this, e.g., to test if analogous inhibitory mechanisms apply to the MutSβ heterodimer viral shuttle (including CoVs, see below).
• *Viral vector vaccines:* As noted, Ambati et al. (2022) suspect that the new gene sequence in SARS-CoV-2 might have arisen in the context of viral research, ostensibly to learn about viruses themselves, as e.g., in the above context. The insights of such an analysis would likely inform the design of novel therapeutics, the main aim of the patent. Thus, in addition to studying CoVs for research purposes in cancer-related pathologies, viruses could have been designed as a vector to deliver specific oncology-related mmRNAs into human cells. The Moderna patent (Bancel et al., 2017) places special emphasis on this step, emphasizing that the novel oncology-related polynucleotide sequence encoding a polypeptide of interest would need to be incorporated into a vector such as plasmids, *viruses,* cosmids, and artificial chromosomes. In this light, certain CoVs may have been engineered as a recombinant vector *vaccine* to express oncology-related genes of interest.

The notion that synthetic mRNAs may help repair the damage done by viral proteins to the host cell immune responses is analogous to that employed for mRNA Covid-19 vaccines: mRNAs specifically designed and introduced into living cells get translated by the host cell machinery which, in turn, is expected to result in the production of the anticipated proteins—with Covid-19, it is the spike antigen of the virus, whereas for therapeutic purposes, it would be key proteins that were compromised by the nuclear viral proteins to support specific immune responses such as DNA repair, or those that inhibit the import of viral proteins, for example. The idea of utilizing synthetic mRNAs as gene-therapy agents to provide missing or defective proteins is not new and had previously been explored for decades (Malone et al., 1989; Wolff et al., 1990) and is one of the main pillars of the Moderna patent (Bancel et al., 2017).

Interestingly, while Ambati and collaborators suspected that the role of MSH3 was to lead to DNA repair deficiency in human cells, MSH3 is itself a DNA repair protein. It acts to recognize mismatch repair and helps to repair double-stranded breaks (van Oers et al., 2014). Furthermore, MSH3 may be a shuttling protein as well, a feature that is highly relevant in this context, as is the discovery of agents that either promote or prevent nuclear import of MutSβ (MSH2-MSH3 heterodimer) which have been investigated to treat trinuleotide repeat expansions that drive Huntington’s disease (HD) and other severe genetic diseases.

### 3.2 An extended experimental framework that could facilitate the recombination of a CoV with an mmRNA encoding human MSH3

The above extends in a hypothetical manner the experimental underpinnings envisioned by Ambati and colleagues. Doing so not only provides a feasible rationale for a joint research objective that aligns CoVs with cancer research and MSH3. It also outlines in which way the postulated viral recombination event could have materialized.

According to the refined framework envisioned here, a genetic recombination event could have led to the FCS insert in SARS-CoV-2 in several ways.

• *Infection of MSH3-transfected cells with viral vector vaccines*: To test recombinant viral vector vaccines carrying a novel anti-tumorigenic gene, it is likely that cell lines prone to tumorigenesis (e.g., those deficient in DNA repair) would have been injected with different variants of a viral vector vaccine (i.e., different SARS-like viruses encoding different therapeutic mmRNAs). Presence of the optimized MSH3 gene (to evoke DNA deficiency) in the cell culture could have led to recombination with the viral vector vaccine (a SARS-like virus) and resulted in the integration of the novel SARS-CoV-2 FCS insert.
• *Testing the therapeutic potential of (modified) MSH3, the control of its intracellular shuttling/localization, and its potential as a cellular defense (which likewise would have relied on infecting MSH3-transfected human cell lines with SARS-like viruses):* Again, this involvement of MSH3 is different than the one envisioned in (Ambati et al., 2022) to evoke DNA deficiency, likely for some research purposes. Alternatively, MSH3 itself may have been examined for its potential to act as a viral protein shuttle/viral defense. This is based on the observation that MSH3 contains Nuclear Localization and Export Signals which in an inflammatory context have been shown to enable nuclear-cytosolic shuttling of proteins (Tseng-Rogenski et al., 2020). A key question that remains is whether the shutting of SARS-like proteins into the nucleus could actually be inhibited. Now, the study of Huntington’s disease (HD) and other expansion diseases has revealed potential therapeutic options via the control of MutSβ localization that seems to be relevant in this regard. Intriguingly, Williams et al. (2020) discovered that the acetylation status of lysine residues in the MSH3 NLS effectively controls the subcellular localization of MutSβ. Of note, this gives rise to specific treatment options that either favor deacetylated MutSβ—which can translocate in and out of the nucleus—or the acetylated form—which prevents nuclear reentry. Given that NLS-driven viral protein nuclear translocation is common in SARS-like infections (Sattar et al., 2023), it would have been reasonably to test whether treatments favoring acetylated MutSβ, or others, could likewise impair nuclear import characteristics of CoVs present in the same cell culture.
• *Testing of viral evolution/escape:* In this hypothesized framework, CoVs could have played three roles: a) For the analysis of genetic features which allow SARS-like viruses to translocate some of their proteins into the host cell’s nucleus; b) The design of CoVs that express novel anti-tumorigenic genes such that attenuated forms thereof could be used as a cancer vaccine; c) SARS-like viruses as the targets of novel drugs which prevent the nuclear localization of their proteins. Even though a recombination event between those viruses and synthetic mRNAs seems feasible in all these scenarios, their odds may be different and significantly increased by specific evolutionary pressure, e.g., when targeting the ability of CoVs as a carrier of therapeutics or when trying to prevent nucleocytoplasmic transport of viral proteins.

As described in Sect. 2.4, evolutionary pressure may be one of the key factors to foster CoV escape mutants via recombination events. In which way this seems relevant to the Ambati et al. hypothesis is further detailed next.

### 3.3 Selective pressure in the lab may have created both a novel FCS and an unintended NLS

The above described several hypothetical ways in which viruses, either during viral or vaccine research, could have unwittingly or intentionally been modified via recombination to acquire the purported patented sequence in the SARS-CoV-2 genome. In some of these cases, this could have been fostered by lab-induced pressure leading to viral evolution.

An interesting aspect related to MSH3 highlighted above is that it recently was recognized as a shuttling protein containing special nuclear localization signals (NLSs) (Tseng-Rogenski et al., 2020). The critical role of novel NLSs in SARS-CoV-2 has only recently become known when it was discovered that this virus has unexpectedly improved nucleocytoplasmic trafficking potentials. Specifically, a recent study by the National Institute of Allergy and Infectious Diseases (Sattar et al., 2023) analyzed novel characteristics of SARS-CoV-2 related to its potential for its proteins to be transported into the nucleus. Notably, Sattar and collaborators found that unexpectedly both the spike (S) protein and mRNA translocate into the nucleus in SARS-CoV-2-infected cells. Even though NLS-driven translocation of some SARS-like proteins is well established, neither of these is as effective as for SARS-CoV-2’s S protein.

The critical observation that SARS-CoV-2 proteins, most notably the spike, can translocate to the nucleus was first shown in (Jiang and Mei, 2021) which, however after its first publication appeared as too controversial since it raised the potential of the same mechanisms to also apply to the spike produced by Covid vaccines. The paper ended up being retracted - albeit, with the findings essentially to be re-discovered by Sattar et al. (2023) who did not seem to be aware of Ref. (Jiang and Mei, 2021).

The goal of Ref. (Sattar et al., 2023) was specifically to measure the extent of subcellular localization of S mRNA and protein. Potential processes explaining the mechanisms of the translocation were in part obtained via machine-learning models, building on the notion described above, i.e., that the viral genome is transcribed in a discontinuous manner. Since S mRNA was seen to colocalize with the S protein, Sattar et al. believe that the nuclear translocation is mediated by a novel NLS in the S protein. Intriguingly, this NIAID study (Sattar et al., 2023) also found that this new NLS motif was present at the polybasic FCS. This was surprising since the specificity of the amino acid motif, a furin cleavage motif, was not expected to also fulfill the characteristics of an NLS motif.

The crucial point here is that the inserted sequence—which above was investigated from the perspective of an FCS—also creates an unprecedented NLS. Specifically, the novel “PRRA” FCS is subsumed within the longer sequence “NSPRRARSV” - with “PRRARSV” being a novel NLS. The astonishing fact is that both of these are functional: the FCS is key to allowing SARS-CoV-2 to infect human cells and the NLS shuttles viral proteins and mRNA, and possibly the whole genome, into the nucleus.

To the Ambati et al. hypothesis, this is significant, because of the following.

• The double NLS/FCS functionality is believed to have drastically enhanced the pathogenicity and infectivity of the new virus; this is in line with natural selection of the fittest CoV genome which, as summarized above, is now believed to be generated and selected by frequent and continuous recombination events.
• It seems feasible that the research aims to examine the nuclear translocation of viral proteins in the context of potential inhibitors as outlined in the previous section, has created the laboratory framework conducive to viral evolution and recombination.
• Specifically, experiments that assessed the fate of CoVs in the presence of the controllable shuttle protein MSH3, agents that can hinder nuclear transport via NLS-acetylation, or established inhibitors of viral nuclear transport such as ivermectin, may have created enough pressure on these viruses that this could have fostered the development and selection of viral mutants that can transport their proteins into the nucleus in some new/improved ways.

In sum, the experiments to develop inhibitors of viral nucleocytoplasmic transport as envisioned in this section could have created substantial pressure on the virus. Escape mutants may indeed have involved novel/improved features for nuclear translocation, e.g., afforded by a novel NLS that is an FCS as well. In this light, the above-described research objectives could explain the recombination event of Ambati et al. (summarized in Table 2). Importantly, even though such recombination may be rare naturally, as has been suggested, under specific experimental settings as postulated above, the extensive evolutionary pressure may have fostered the survival of exactly those rare viral escape mutants with such a unique insert encompassing the FCS.

**TABLE 2:** While the genetic recombination of a CoV with an RNA described by Ambati et al. may happen rarely in a natural context, the above argues it may be realized in a certain laboratory setting as particularly fostered by specific evolutionary pressure during the testing of novel therapeutics.

Type	Arguments objecting to/supporting the recombination event postulated by Ambati et al.
Cons	• The viral recombination of a CoV with a synthetic RNA leading to a certain insert in SARS-CoV-2 as postulated by Ambati et al. requires two template switching events
	• Template switching has been extensively studied. For example, with CoVs, it is known that a major regulator of template switching is the amount of base pairing in the donor and acceptor (Zúniga et al., 2010; Sola et al., 2011)
	• For the new sequence insertion as reported by Ambati et al. (19 nucleotides), it is expected that the frequency of the crossover events would be extremely low because the two crossover events would have to be very close together
Pros	• MSH3 is involved in double-strand break (DSB) repair via homologous recombination (Tseng-Rogenski et al. (2020) and references therein) and it seems to be a shuttling protein itself. Due to its involvement in Huntington’s disease (HD) and related human genetic diseases, the control of the subcellular localization of MutSβ (MSH2-MSH3 heterodimer) has been pursued as a novel therapeutic opportunity. Treatments that favors acetylated MutSβ allow it to exit the nucleus but hinder its nuclear reentry.
	• As detailed above, a core pillar of Moderna’s cancer research may have been to target the nuclear transport of CoV proteins, a viral feature that is known to disrupt DNA repair. It is reasonable to envision an experimental context wherein MSH3 was tested to a) better elucidate the role of CoV nuclear import and its role in cancer, and b) try to exploit the therapeutic potential of controlling MSH3 localization and mechanisms that inhibit MutSβ nuclear import (notably, NLS acetylation), to impair NLS-enabled viral translocation of SARS-like viruses. By its very nature, such experiments could have created substantial evolutionary pressure on a CoV, fostering the development of escape mutants with improved nuclear transport profiles
	• Recombination between CoVs plays an important role in CoV evolution as it can alter host range, pathogenicity, and transmission patterns. Contrary to previous results that identified RNA structure and sequence identity as the major regulators of recombination in RNA viruses, more recent studies have shown that recombination is a promiscuous event that is significantly influenced by evolutionary mechanisms and selection processes (Alnaji et al., 2022; Wang et al., 2022)
	• For natural genetic viral recombination, their heritability is mediated by the replication fitness of the resulting progeny genome (Graham et al., 2018). However, evolutionary pressure has recently been recognized as a key factor dictating both the selection and maintenance of recombination events in RNA viruses (Bentley and Evans, 2018; Alnaji et al., 2022; Wang et al., 2022)
	• The influence of evolutionary pressure in the lab has not been sufficiently studied to fully understand, let alone eliminate, the potential of RNA viral recombination in such settings
	• In this work, particular experiments are outlined that logically make sense in the context of the Ambati et al. hypothesis. It is suggested that the resulting specific evolutionary pressure on some CoVs may have been in tandem with the development and survival of escape mutants harboring the novel NLS/FCS sequence - which is the core part of the insert indicated by Ambati et al
	• Thus, certain laboratory experiments as explained herein could have favored the genesis and heritability of the recombination events as postulated by Ambati et al

## 4 Special considerations for biosafety and biosecurity

A first goal of this article was to scrutinize the feasibility of the postulated mechanism by Ambati and collaborators and to envision specific laboratory settings that could increase the odds of such events. The Ambati et al. postulate, covering only the FCS, cannot resolve the viral origin question *per se*. Nevertheless, the evidence developed above regarding the implicated genetic recombination events points to the existence of biorisks which have not been sufficiently appreciated, especially for their potential for future malicious exploitation.

### 4.1 Gaps in existing biorisk regulation

Biorisk management has long been divided into biosafety and biosecurity, where, informally, the former targets accidental/unintentional vulnerabilities and the latter, deliberate ones^8^. It has been recognized that whilst biosafety and biosecurity are inextricably linked, they are governed by different legal, policy, and regulatory regimes. Albeit, “[b]oth aim to keep dangerous pathogens safely and securely inside the areas where they are used and stored … ” (National Research Council, 2015).

Over the years, risk assessors have known that regulation has been vastly complicated by the nomenclature related to DU, DURC, and GoF. Also, it has become increasingly clear that because of new technologies, societal issues, and others, many facets are incompletely understood, allow different interpretations, and that risk assessment is not free from subjectivity either (Bulletin of the Atomic Scientists, 2023). The above, while it intersects biosafety and biosecurity, falls outside existing regulations, because of the following.

#### 4.1.1 Beyond stated pathogen/biological weapons research

Existing biorisk policy, legislation, and regulatory guidelines focus on agents which from the outset suggest some hazardous potential (e.g., ‘biological agents and toxins,’ ‘pathogens,’ ‘bioterrorists,’ ‘bioweapons’). Apparently triggered by the Covid pandemic, we now see intense global efforts with an increased focus on pathogen research^9^. However, the above raises concern that specific components of research with rather different objectives, including those that certainly would be classified as benevolent, may converge to harbor under-appreciated GoF/DURC vulnerabilities, raising the prospect of the criminal genesis of dangerous pathogens in clandestine.

#### 4.1.2 Infeasibility to calculate biorisk

While traditionally biorisk policy has focused on the likelihood and potential impact of a range of risks (The Royal Society and the International Council for the Life Sciences, 2009), requiring both biosafety *and* biosecurity (National Research Council, 2015), the above highlights several challenges in doing so. Without awareness of the discussed vulnerabilities, their feasibility and consequences have been under-appreciated and there are no mitigation measures in place, especially against deliberate misuse. The general lack of security-by-design and by-default of the underlying technologies leads to the potential for a crime harvest, so that the above mechanisms or routes to harm could be exploited as a Trojan horse in the form of novel exploits that are largely unpredictable.

#### 4.1.3 A blurring of biosafety and biosecurity

Above, it was argued that certain experimental conditions may result in various viral recombination events with a range of outcomes. Nonetheless, the implicated biorisks may not fall into a clearly defined category such as “accidental” *versus* “deliberate,” and the same applies to potential actors. Biological risk itself comprises a spectrum, ranging from unintended/accidental to targeted malicious misuse, and encompasses naturally occurring diseases, re-emerging infectious diseases, unintended consequences of research, laboratory accidents, lack of awareness, negligence, and deliberate misuse (The Royal Society and the International Council for the Life Sciences, 2009).

Thus, a binary distinction between ‘unintentional’ and ‘deliberate’ may be difficult, even more so as synthetic biology has increasingly utilized digital technologies (e.g., cloud, mobile, cyber-physical/biological systems). In fact, in (Mueller, 2020), I first argued that in such contexts, the notions of safety and security cannot be readily separated, and this dilemma is further exacerbated by the convergence of fields, knowledge gaps, DU interpretations, and the insurmountable inherent gap between biology, computerized technology, and web interfaces.

### 4.2 Potentials for a crime harvest and related dangers

Risk assessment of dangerous organisms and pathogens has stressed the importance of taking into account their weaponization potential, the capability (including both scientific knowledge, tacit knowledge, and technological know-how) and intent of an adversary, and the potential consequence of an intentional release or misuse (National Research Council, 2015).

However, a major difficulty to quantify criminal or terrorist risk has been described via the limited historical precedent of biological weapons misuse (The Royal Society and the International Council for the Life Sciences, 2009; National Research Council, 2015). Key factors in this regard, including ‘expected outcome,’ ‘feasibility of attacks,’ and ‘motives,’ align with those made by the information-security community (Mueller and Barros Lourenco, 2023) - which over the decades has gained extensive experience with intentional forms of crime. Below, these will be specifically analyzed in the context of RNA recombination as discussed above.

#### 4.2.1 Factors that increase the potentiality of misuse

Whilst the majority of the life science community is highly conscientious, under-appreciated risks such as the above have not received much attention, especially from a security perspective. Table 3 summarizes key aspects that can drive criminal exploitation of these new vulnerabilities.

**TABLE 3:** Factors that increase the misuse potential of the type of research indicated by Ambati et al. - and extrapolated herein to highlight the feasibility and danger of these unrecognized vulnerabilities.

Key factor	Comments
Existing policies have cautioned not to over-emphasize hazards and threats, especially downplaying security concerns	The notion that laboratory work in general could be maliciously exploited, has long led to the sentiment to not create unsubstantiated public fear. For example
	• The Royal Society and the International Council for the Life Sciences (2009) cautioned in 2009 that “It is also important not to over emphasise one particular risk, such as terrorism, which can undermine public confidence in risk assessments of the range of hazards and threats.”
	• The same sentiment is ongoing, as demonstrated by the fact that there is relatively little published work that analyzes what threat actors could learn from the Covid pandemic
Drug and vaccine R&D has not received adequate scrutiny for their potential to be misused by threat actors	As increasingly seen since the Covid pandemic, suggestions that certain research objectives like those discussed above could be misused, have not been widely appreciated
	• Any such suggestions may quickly be (mis)understood as implying culpability of certain companies related to past events
	• The very notion that vaccines or viruses could be turned into harmful agents has essentially been regarded as ‘verboten,’ out of fear of political, sociological, or other detrimental consequences to science (Andersen et al., 2020)
	• As before, this very climate and gap in biorisk awareness has created an unprecedented security vulnerability
Zero-day exploits	The feasibility of malign or criminal use of genetic recombination in the context of viral and vaccine research has not been sufficiently recognized
	• This is likely because these applications are inherently seen as being developed with a beneficial objective and with the common goal to save lives and improve the health of humans
	• The underlying biosafety challenges have prompted R&I into preventing accidents and unintended outcomes, albeit at the expense of targeting criminal aspects
	• The convergence of these factors may support a crime harvest and provide substantial advantages to those intending to harm
Challenges with attribution (historical experience)	The ongoing struggles to clearly prove the origin of SARS-CoV-2 can inform future criminals. A lack of attribution has long been recognized as a significant driver for misuse (Murch, 2015)
A general difficulty to distinguish natural from deliberate events	Ironically, whilst synthetic biology strives to mimic nature to enhance the safety and efficacy of bioengineered products, this very same feature may also facilitate misuse
	• Specifically, synthetic genetic material, as it can, and does, play roles similar to its natural counterpart, can therefore become highly attractive for bad actors
	• The very indistinguishability between ‘natural’ and ‘engineered,’ may enable threat actors to infiltrate cell lines not only via contaminants but also allow criminal work to be done in secret and additionally fostered by insecure technologies
	• The fact that both positive and negative strand RNAs may play critical roles, would further complicate analysis and detection
The need for tacit knowledge may be minimized	Whilst traditionally, building a bioweapon has relied on intense tacit knowledge and skill, and would have required access to very specific and expensive technology and devices, these constraints are challenged by some of the above
	• Given that recombination does naturally occur between RNAs and CoVs, this may assist bad actors and minimize the skill they need
	• Exploiting the tendency of RNAs to recombine, bad actors may therefore resemble someone with a match in a dry forest
	• When done covertly, this may be able to facilitate (some) recombination events without needing to employ molecular specifics
Sequence homologies have not been sufficiently scrutinized for their potential for misuse (camouflaging, covert ingression, etc.)	Special features identified above are particularly amendable to malicious exploitation with adverse clinical consequences
	• CoV recombination itself has long been known to play an important clinical role as it can change host/tissue range, increase infectivity and pathogenicity of viruses, and lead to vaccine escape
	• The substantial amino acid sequence matches between CoVs and humans can have profound adverse clinical sequelae. For instance, Harrison and Sachs. (2022) found that SARS-CoV-2’s FCS also exists in the *α* subunit of the human epithelial sodium channel ENaC where it is functional. This “molecular mimicry” between the viral FCS and that of the human ENaC leads to, 1) a detrimental competition for host furin and decreased expression of ENaC related to its ion channel function which is known to compromise airway function, and 2) cross-reactivities of antibodies with human ENaC from SARS-CoV-2 infection, a factor implicated with severe forms of COVID-19
	• In addition to a covert insertion of an FCS, the analogous malicious exploitation of molecular mimicry between other mammalian/human and viral proteins may lead to the disruption of the balance and kinetics of critical host enzymes, auto-antibody development, and other adverse events
Several convergence issues creating knowledge gaps	The traditional biosecurity landscape is substantially increased by a convergence/blurring of
	• Research objectives, experimental underpinnings, and potential pathways of harm
	• Biotechnology with ICT technology, which creates a vast array of novel cyberbiosecurity gaps (Jean et al., 2018; Murch et al., 2018; Murch and DiEuliis, 2019; Mueller, 2020; Mueller and Barros Lourenco, 2023)

#### 4.2.2 Susceptibility and outcome

The current lack of rigorous cyber-biosecurity risk management practices and a poor security mindset have made the entire biotechnology sector vulnerable to exploitation. For example, according to a Forbes article^10^, pharma and biotech companies are affected by more cybersecurity breaches than any other industry, with some of the high-profile attacks in recent years involving espionage and intellectual property theft related to COVID-19 vaccine development and attacks on technology involving DNA sequencers. Security risk analyses are also plagued by sociopolitical influences as demonstrated by the ongoing debates involving the pandemic origins and what this means for future events. While there is no sound rationale that the pandemic was deliberately initiated, the numerous controversies may in fact inform bad actors (Mueller, 2023).

The novel vulnerabilities depicted above include cancer research, drug development, and viral research, which constitute highly lucrative assets whose compromise can have systemic implications with enormous social, health, and economic sequelae (further detailed in Table 3).

#### 4.2.3 Actors, motives, and capability

Bad actors may, in addition to gaining physical access to laboratory processes or devices, also mount their nefarious activities by exploiting gaps that are facilitated by the convergence of the underlying technologies (Jean et al., 2018; Mueller, 2020). Both factors combined increase the attack surface to realize viral recombination events as discussed above, via the covert disruption of confidentiality, integrity, and availability (CIA triad) of cyber-physical and bio-related processes, for example, through the swapping of biological/chemical/physical entities and/or their digitized description, mislabeling, masquerading, or other camouflaging attacks, including those fostered by the interrelationship and gap between computerized/automated descriptions, applications, web interfaces, and the actual entities (devices, processes, biomatter, etc.), ranging from research and planning, across the supply chain, to the final biological/bioengineered outcome in question.

Related work on cyberbiosecurity by The European Union Agency for Cybersecurity (Mueller and Barros Lourenco, 2023) has identified key motives that can drive attacks in the life sciences as they are fostered by computerized and networked technologies which are extended to the present context of viral recombination in Table 4.

**TABLE 4:** The motives, mechanisms, and potential outcomes of the possible pathways of harm discussed herein have not previously been analyzed from a biosecurity perspective and are not covered by existing policy and regulation.

Motives	Description and potential outcome
Criminal/for profit	Bad actors could ingress synthetic RNA contamination, which under certain laboratory conditions may enable genetic recombination of RNA viruses. Specific aims of such criminal acts may be to
	• Derail competitor’s research programs (e.g., involving viral or oncology-related research) via unrecognized genetic recombination events
	• Create new viruses and blame a competitor for the conducting of forbidden GoF work
	• Corrupt competitor’s manufacturing of vaccines or therapeutics
Bioterrorism	Covert/disguised genetic recombination events may be employed for the design of harmful viruses for their actual, staged, or threatened employment as a bioweapon
	• Traditionally, biosafety policy has placed great emphasis on preventing the design and manipulation of pathogens with pandemic potential (PPPs)
	• Focus has been on specific adversaries that are believed to be interested in creating bioweapons
	• Due to a) a lack of significant historical events related to state actors and b) limited dangers seen from non-state actors (i.e., extremists with apocalyptic ideology or sociopathic tendencies or rare mentally ill insiders (The Royal Society and the International Council for the Life Sciences, 2009)), biosecurity has not been regarded as the most imminent threat related to the emergence of PPPs
	• Traditionally, the view has been that the dangers of PPP are mostly caused by zoonosis
	• All the above ignores new technological developments, which could a) enable threat actors to utilize automated processes/AI to optimize laboratory settings which increase the odds of viral recombination, b) exploit the lack of existing security-by-design and by-default of underlying technologies, and c) realize their intrusions at various points of the largely unsecured threat landscape of modern biotechnologies (Mueller and Barros Lourenco, 2023)
Insider attacks	• Insider threats have always played an important role in various security contexts, comprising a range of nuances and motivations including accidental and malicious (Mueller and Barros Lourenco, 2023)
	• Insiders often have direct access to relevant (biological) material, devices, and processes, which may allow the covert infiltration of genetic contaminants, particularly as these are difficult to spot
Circumventing GoF policy	• Given that life-science researchers have always been conscientious, albeit overall lacking a security mindset, dangerous research projects camouflaged as benign might not readily be detected
	• The ongoing controversy as to what type of pathogen research is necessary (“good”) vs what is too risky (“bad”) has created a gap in clear and uniform biorisk assessment and policy
	• Disparate views, interpretations, and different policies in distinct jurisdictions may increase the likelihood that certain work be hijacked
DU controversies and a new type of ethics-based hacking	By their very nature, DU issues comprise two sides and it may not always be easy to distinguish “good” from “bad.” Ironically, this inherent dilemma, which has the potential to significantly polarize scientists and policymakers, may also create a new type of (ICT-based) attackers who feel their views are not adequately appreciated

### 4.3 A criminal context may turn things on their head

Traditionally, biorisk adversaries have been limited to specific groups with extensive skill and interest in creating bioweapons. However, the above vulnerabilities may be susceptible to a larger group of actors, requiring less know-how and tacit knowledge for their exploitation (Table 3; Table 4). Notably, actors could aim to facilitate interactions between the man-made world (e.g., synthetic RNAs) and the ‘living’ world (e.g., viruses) without aiming for a specific outcome. In the context of drug or vaccine development, viral recombination events can significantly impair research outcomes and product quality and derail a competitor, even if the adversary cannot target a particular type of recombination with specific RNAs.

Secondly, in addition to just waiting for a chance outcome, which could be fostered by covert ingression of RNA contaminants for instance, bad actors may even benefit from biorisk analyses which may expose which determinants could increase the likelihood or scope of a specific outcome. In this sense, information that may be regarded as useful to facilitate benevolent R&D may have an unrecognized DURC component nonetheless. For example, insights derived from the development of recombination-resistant CoVs for live-attenuated vaccines (Graham et al., 2018), may inadvertently also reveal factors that increase the odds of viral recombination.

More generally, a biosafety analysis that identifies where and how risk is most effectively targeted may likewise inform bad actors, revealing where a successful attack could provide “the greatest bang for the buck.” In this light, it is unclear how to align biosafety risk mitigation with security principles without providing exploitable information (“side channels” (Mueller and Barros Lourenco, 2023)) and pointing bad actors to unrecognized DURC potentials, weak spots, or most attractive targets.

## 5 Conclusion

This work envisioned a hypothetical framework that enables underappreciated vulnerabilities of CoV recombination in a lab, and which, at least theoretically, could have led to the integration of the SARS-CoV-2 FCS. Specifically, this article identified several uncertainties that arose in the context of the Ambati et al. controversy and found several gaps in current biorisk assessment and policy (summarized in Table 5) which could inform future threat actors.

**TABLE 5:** An analysis of the controversial Ambati et al. postulate regarding the integration of a sequence encompassing the SARS-CoV-2 FCS has identified critical gaps which should be a key priority for synthetic biology risk assessment, especially from a criminal perspective.

Category	Main finding
At the sequence-analysis level	• Genetic changes may lead to multiple and unexpected biological mechanisms, as seen here with the double FCS/NLS functionality
	• It is possible that dangerous sequences (e.g., here the FCS) are, via their reverse complements, characterized as benign (here, the MSH3 gene)
	• Biorisk assessment is complicated by unknown reading frames and reverse complement sequences which can allow dangerous sequences to be obscured
	• The potential for criminal exploitation of such dangerous sequences has not been adequately appreciated
	• Given that the likelihood of finding matches and sequence homologies is high, as shown in the Commentary by Dubuy and Lachuer (2022) to Ref. (Ambati et al., 2022), this creates a largely underappreciated biosecurity vulnerability to camouflage dangerous sequences
	• Short genetic sequences can lead to erroneous interpretations when a) it appears there is a homology between sequences - implying relationships of organisms - that is artefactual and simply by chance, or when b) true homologies involving the negative sequence are not readily recognized
	• This has critical implications for well-intended research programs to be hijacked and diverted into covert bioweapon development programs
Research objectives and goals	• Various apparently distinct research objectives and goals with intrinsically benign features may lead to a convergence with unique DURC potential
	• Synbio products such as synthetic RNAs may be able to interact with the man-made and the natural world in ways that have not been sufficiently appreciated
	• In recent years, research has shown the increasing role of host immunity, evolutionary pressure, genome function, and viral fitness as key factors driving the genetic recombination of positive-strand RNA viruses
	• Even if specific recombination events are deemed unlikely to arise in nature, this does not mean that the same could not be intentionally targeted in clandestine by lab-imposed evolutionary pressure
	• Since genetic recombination of viruses contributes substantially to the emergence of new viral lineages, expansion in host tropism, adaptations to new environments, increased virulence and pathogenesis, and escape to vaccination, it seems plausible that the development of more dangerous viruses through recombination with synthetic RNAs is substantially enhanced in a susceptible laboratory environment
Risk spectrum and assessment	• It is imperative to consider risk management across the full risk spectrum, also regarding novel actor types and motives. In addition to unintentional risks, the potential for deliberate misuse may extend beyond more traditional GoF/DURC scenarios and traditional bioterrorists
	• Risk management that identifies where and how risk scales most rapidly, e.g., in certain “high risk” or otherwise susceptible experimental contexts or with increased use of technology (Heinemann et al., 2021), may inevitably inform bad actors, who may thereby learn critical information about vulnerabilities, weak spots, and most attractive targets
	• A failure to appreciate emerging attack potentials fostered by the convergence of new ICT-based technologies and under-appreciated molecular mechanisms may enable the deployment of nefarious “Trojan horses,” especially if nobody suspects them

It has been suggested that the odds of the particular RNA recombination indicated by Ambati et al. may be low as this would require two crossover events very close together. Nonetheless, recent research about the recombinability of RNA viruses stresses the foundational role of evolutionary pressure in both the selection and maintenance of viral recombination events, a factor that is of great relevance in a lab environment. Therefore, due to the convergence of particular research objectives and experimental conditions as postulated above, the type of recombination as envisioned by Ambati et al. cannot be ruled out, particularly in a criminal context.

**FIGURE 2:**
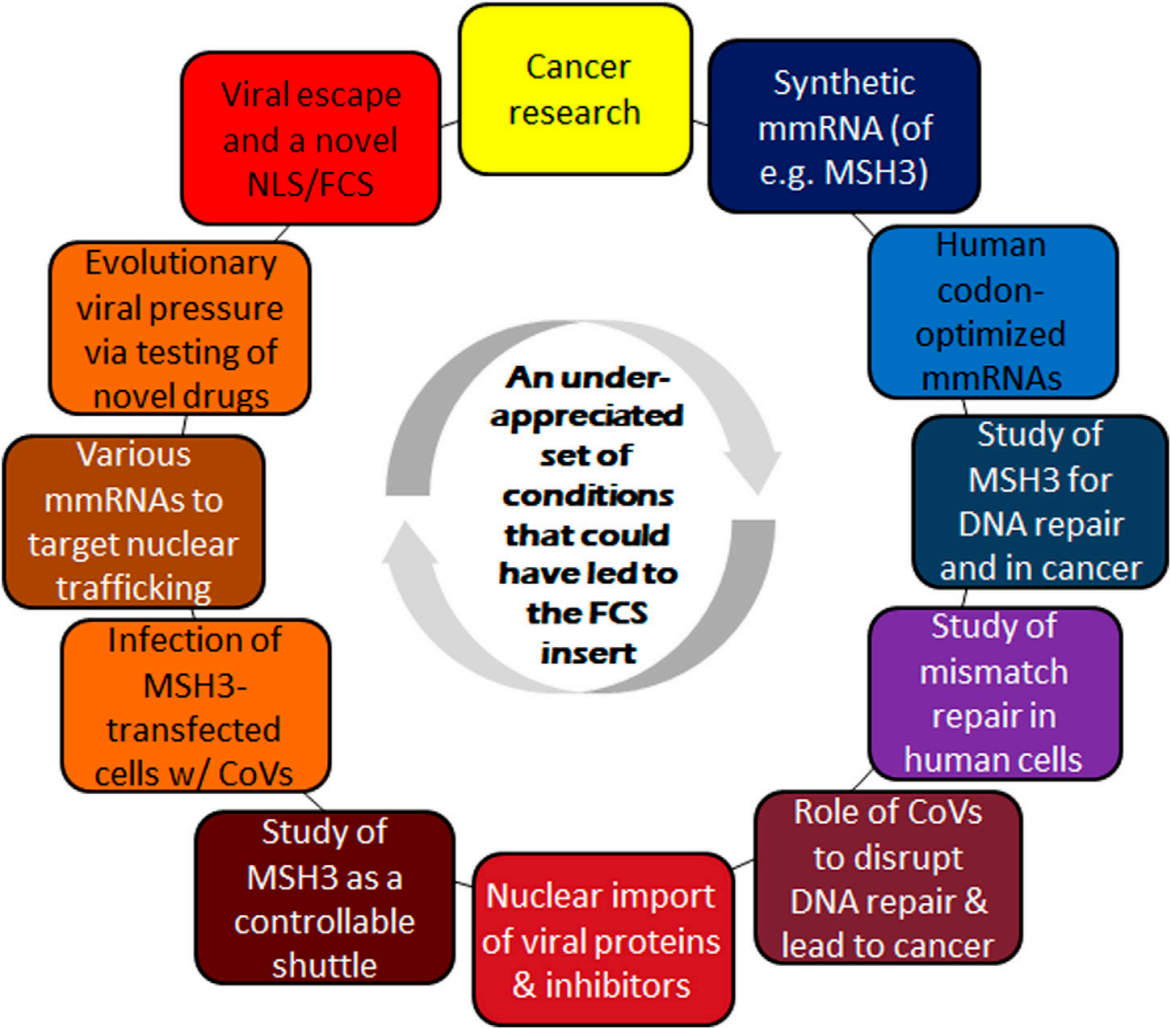
Postulated interrelationship/convergence of seemingly unrelated research orientations. Ambati et al. (2022) focus on the unexpected occurrence of a patented sequence in the SARS-CoV-2 genome and offer some ideas of what type of experiments could have led to the purported RNA integration. From the outset, it seems difficult to envision under which circumstances the different constituents postulated by Ambati et al., ranging from research involving SARS-like viruses to cancer research, could have converged in a unifying set of experiments to allow the required molecular events to happen. Even though Ambati and collaborators believe this may have been facilitated by a laboratory accident or a deliberate act, the odds of the implied viral recombination event may have been rather small. To address these issues, a rational approach was taken to show that it could have been possible nonetheless. Several hypothetical aspects and scenarios were envisioned that could have combined various seemingly disconnected research orientations and which could also have substantially increased the odds of the specific viral recombination event as postulated by Ambati et al. The figure summarizes the main pillars of this hypothetical framework.

From the outset, it seems difficult to see how the research settings implicated by Ambati et al. could align with the Moderna patent and result in the necessary laboratory experiments to facilitate the hypothesized viral recombination. Whilst an inadvertent or intentional act may still have been possible, the chance of the particular viral recombination may have been rather low. To address this, above, the research objectives implied by Ambati et al. were further refined. A logical rationale was developed for how individual goals, ranging from cancer research, viral vector vaccines, and CoVs, to new oncology-related therapeutics, could have converged into one laboratory objective and set of experiments (summarized in Figure 2).

As detailed above, a core pillar of Moderna’s cancer research may have been to target the nuclear transport of CoV proteins, the latter of which is a well-established pathway to disrupt DNA repair. Given that MSH3 is involved in double-strand break repair via homologous recombination, is able to facilitate nuclear-cytosolic shuttling of proteins, but can also induce DNA repair deficiency when over-/underexpressed, it is reasonable to envision an experimental context wherein MSH3 was tested to a) better elucidate the role of CoV nuclear import and its role in cancer, b) test drugs that prevent the import of viral proteins, and c) specifically target MSH3 for clinical applications. Indeed, only recently, Tseng-Rogenski et al. (2020) speculated that MSH3 could shuttle into the cytoplasm as a part of cellular defense mechanisms to detect invading pathogens that contain DNA and noted the necessity of further studies of this finding. With MSH3, the right concentration and cellular localization are critically important and aberrations lead to severe forms of disease (Tseng-Rogenski et al., 2020). Interestingly, blocking of the MSH3 import function happens via acetylation of its inherent NLS which has been identified as a molecular toggle in broader contexts (Williams et al., 2020).

Essential to the framework hypothesized above is the link between CoV infection and cancer development, and how this could have been targeted by modified mRNAs (which may have included mRNA acetylation as well). Based on the shuttling properties of MSH3 and its putative role in cellular defense, it is feasible to assume that modified MSH3 has been studied as a potential agent to prevent the nucleocytoplasmic trafficking of CoVs. Therapeutic agents that have shown to direct MSH3 shuttling (Williams et al., 2020) may have had direct impact on NLS-driven nuclear trafficking of CoVs as well. Likewise, efficacy testing of select agents such as ivermectin or novel drugs developed to impair the nuclear translocation of CoVs may also have created substantial pressure on these viruses, favored the development of escape mutants with improved nuclear transport profiles, and specifically led to viral mutants harboring SARS-CoV-2’s unique NLS/FCS insert.

With CoVs in particular, viral escape has long been known to be either a mutation- or recombination-driven process, a fact that is demonstrated by numerous research efforts that aim to render live-attenuated CoV vaccines recombination refractory (Graham et al., 2018). Given that naturally, the heritability of a recombination event is mediated by the replication fitness of the resulting progeny genome (Graham et al., 2018) and that more recently, selection and fitness have been regarded as key in recombination (Bentley and Evans, 2018; Alnaji et al., 2022; Wang et al., 2022), it is likely that the same applies to the laboratory-induced selective pressure during the analysis of viruses described above which could have led to the insertion of both an NLS and an FCS.

The notion that the insert surrounding the SARS-CoV-2 FCS could have resulted from laboratory recombination, even though naturally the two required crossover events may be regarded as happening with low frequency, is also in line with the observation that the FCS itself has been shown to appear in steps during serial passaging, as known particularly for the H5N1 flu virus^11^. Therefore, as for general drivers of pathogenicity, those dictating recombination in a laboratory environment are likely governed by different timelines than those known for viral evolution in the wild.

**FIGURE 3:**
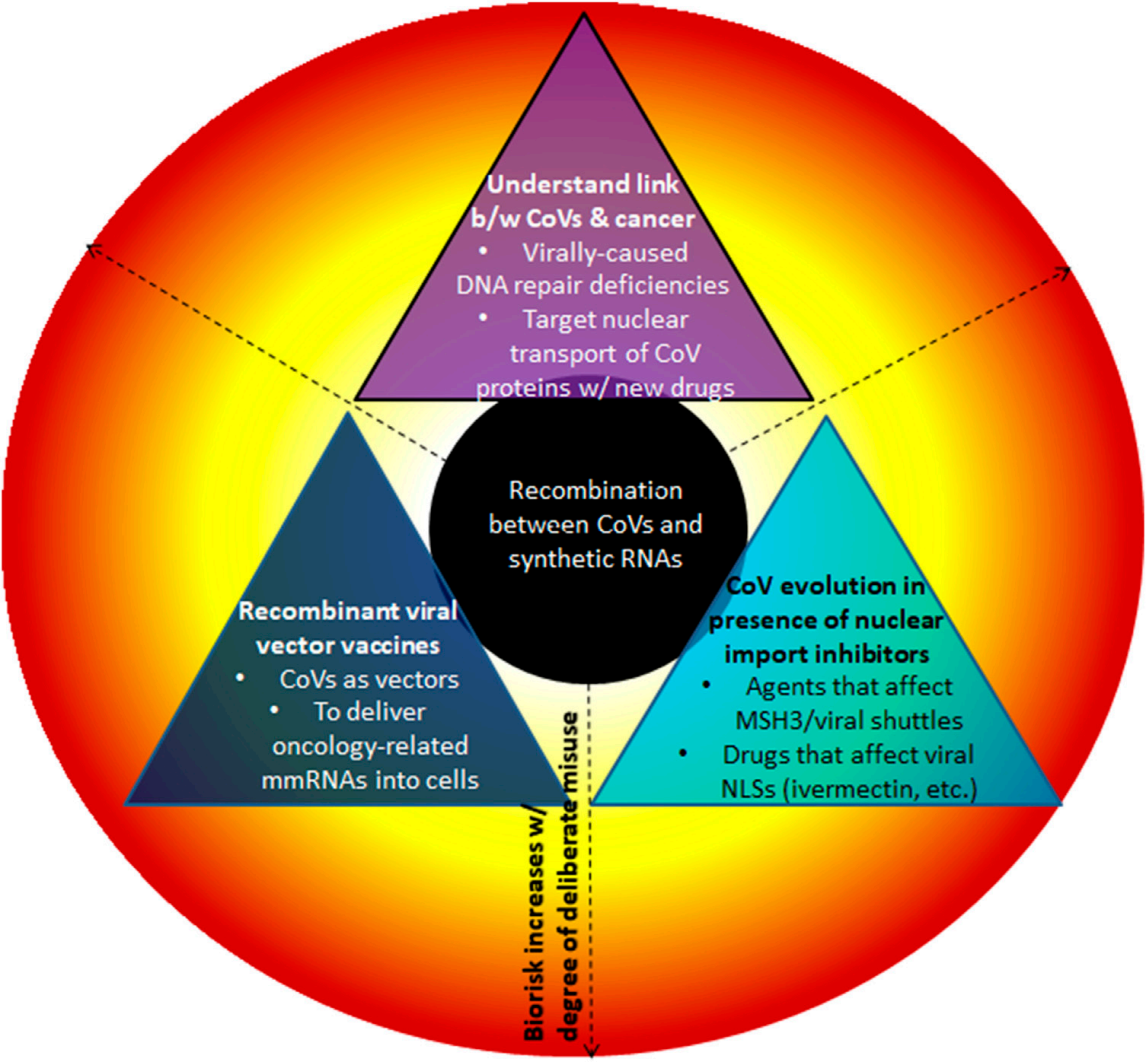
Main research orientations fostering CoV recombination in a laboratory context as motivated by the purported presence of a proprietary sequence in SARS-CoV-2. This analysis shows that there are indeed several ways in which the core postulate by Ambati et al. could have been realized in a laboratory setting. Above, it was argued that recombination between SARS-like viruses and other RNA could have happened via three main types of research experiments, and where MSH3 could be involved, either as a positive control, novel therapeutic agent, or contaminant: 1) Experiments to better elucidate the various DNA repair pathways potentially compromised by nuclear CoV proteins and their role in cancer, 2) The development and testing of the therapeutic potential of synthetic mRNAs as gene therapy agents to mitigate the harmful effects of nuclear import of specific CoV proteins, including delivery vehicles to bring these into human cells. 3) Testing and assessment of CoV evolution and escape in the presence of the tested cancer therapeutics/antivirals. MSH3 may have been of special interest as it is itself a DNA repair protein that may also shuttle into the cytoplasm as part of a cellular defense mechanism, and since the shuttling of MSH3 is controllable via (de)acetylation of its intrinsic NLS. Modified MSH3 or drugs that target NLS-based nucleocytoplasmic trafficking of CoVs could have fostered the evolution and escape of viral mutants with a novel NLS/FCS and improved nuclear transport profile.

In conclusion, Figure 3 summarizes various circumstances envisioned above that *could have* favored the type of viral recombination as postulated by Ambati et al., and which constitute an unrecognized biorisk for future events. The fact that these fall outside of existing GoF/DURC biorisk regulation has critical implications for their potential for deliberate misuse. Even though individual research objectives by themselves may be seen as low risk, convergence repercussions can engender substantial biorisk that may be highly vulnerable to intentional disguise, camouflaging, covert infiltration of contaminants, swapping of biological material, and other crime types.

Recombination plays important roles in the spread, virulence, pathogenesis, and vaccine escape of viruses; for instance, it has been found that the emergence of novel CoVs with enhanced virulence can be explained by recombination events (Graham et al., 2018). Thus, regardless of whether the novel insert in SARS-CoV-2 is the result of recombination as indicated by Ambati et al., the analysis above strongly suggests that bad actors could try to facilitate viral recombination events for various nefarious purposes.

Even though the above shows the *feasibility* of the emergence of the FCS through research projects that are not regarded as risky, this analysis was not done to suggest this is what actually happened, nor was it done to imply Moderna’s culpability in terms of conducting experiments that led to the Covid pandemic. Indeed, the focus of the above was the insert encompassing the FCS alone - which is not the only feature that distinguishes SARS-CoV-2 from its closest relatives, as demonstrated by the additional large number of small sequence differences scattered throughout the genome. Although some may wonder if an adversary could have introduced these on purpose, this seems unlikely. While it is true that the generation and genetic modification of CoVs via synthetic genomics platforms have long been possible (Almazán et al., 2000; Boyd et al., 2000; Thiel et al., 2001) using viral isolates, cloned viral DNA, clinical samples, or synthetic DNA, and even though an improved reverse-genetics platform has enabled the rapid reconstruction of SARS-CoV-2 in only a week after receipt of the synthetic DNA fragments (Thao et al., 2020), the unparalleled tragic toll of this virus on everyone worldwide does not support the idea that it was intentionally made and released from a lab.

The above vulnerabilities cannot be resolved by one overall policy framework and governing authority alone as it seems impossible to envision all possible routes to harm (accidental or deliberate) in all possible contexts. While synthetic biology holds the promise to be able to fully predict and control the outcome, the risks, and dangers described here should be an eye-opener as to how little we still know about the (misuse) potentiality of the generated/modified biological products to interact with the rest of the world, or even change nature itself.

The convergence of technologies and disciplines shows it will be imperative to appreciate the most important pillars of science, skepticism, curiosity, and trans-disciplinary knowledge, and foster a change of consciousness that emphasizes the responsibilities and powers of expertise, insights (including intuition), transparency, and commitment of every researcher and organization involved, to effectively help protect the future of humanity and nature in general.

## Data Availability

The original contributions presented in the study are included in the article, further inquiries can be directed to the corresponding author.

## Footnotes

1
https://www.washingtonpost.com/investigations/interactive/2023/virus-research-risk-outbreak/?itid=hp-top-table-main_p001_f001&mc_cid=1aed65cce5&mc_eid=6cb23747ff

2
https://www.marshall.senate.gov/newsroom/press-releases/sen-marshall-releases-bombshell-covid-19-origins-report/

3
https://www.phe.gov/s3/dualuse/Pages/GainOfFunction.aspx

4
https://www.youtube.com/watch?v=g4rF91BeSJU

5 A detailed list of relevant DURC research criteria can be found in (Godbold et al., 2023).

6 I thank one of the reviewers of an earlier version of this article for this important observation.

7 Throughout, Moderna is taken as a proxy for relevant biomedical stakeholders across academia and industry simply because of the related Moderna patent and not to imply their involvement in the genesis of SARS-CoV-2.

8 More precisely, “Biosafety provides policies and practices to prevent the unintentional or accidental release of specific biological agents and toxins, whereas biosecurity provides policies and practices to prevent the intentional or negligent release of biological materials or the acquisition of knowledge, tools, or techniques that could be used to cause harm,” (National Research Council, 2015).

9 e.g., https://thebulletin.org/pathogens-project/,https://www.who.int/news/item/26-04-2023-who-launches-new-initiative-to-improve-pandemic-preparedness

10
https://www.forbes.com/sites/forbesbusinesscouncil/2021/03/18/how-the-pharmaceutical-industry-can-secure-networks-to-avoid-cyberattacks/?sh=2a5bffdb1eb3

11
https://theintercept.com/2023/01/19/covid-origin-nih-emails/

## Abbreviations

CoV, coronavirus; DDR, DNA damage response; DU, Dual-Use; DURC, dual-use-of-concern; ENISA, The European Union Agency for Cybersecurity; FCS, furin cleavage site; GoF, gain of function; MSH3, The DNA mismatch repair protein MutS Homolog 3; NLS, nuclear localization signal; SARS-CoV-2, Severe acute respiratory syndrome coronavirus 2.
